# Description of inter-institutional referrals after admission for labor and delivery: a prospective population based cohort study in rural Maharashtra, India

**DOI:** 10.1186/s12913-017-2302-4

**Published:** 2017-05-19

**Authors:** Archana B. Patel, Amber Abhijeet Prakash, Camille Raynes-Greenow, Yamini V. Pusdekar, Patricia L. Hibberd

**Affiliations:** 1 Lata Medical Research Foundation, Nagpur, India; 2 Sydney School of Public Health, Sydney Medical School, The University of Sydney, Camperdown, New South Wales Australia; 3 Boston University School of Public Health, Boston, USA; 4 Boston University School of Medicine, Boston, USA

**Keywords:** Maternal referral, Inter-institutional transfer, Stillbirths, Neonatal mortality, India

## Abstract

**Background:**

In 2008, the Indian government introduced financial assistance to encourage health facility deliveries. Facility births have increased, but maternal and neonatal morbidity and mortality have not decreased raising questions about the quality of care provided in facilities and access to a quality referral system. We evaluated the potential role of inter-institutional transfers of women admitted for labor and delivery on adverse maternal and neonatal outcomes in an ongoing prospective, population-based Maternal and Newborn Health Registry in Central India.

**Methods:**

Pregnant women from 20 rural Primary Health Centers near Nagpur, Maharashtra were followed throughout pregnancy and to day 42 post-partum. Inter- institutional referral was defined as transfer of a woman from a first or second level facility where she was admitted for labor and delivery to facility providing higher level of care, after admission to the day of delivery. Maternal mortality, stillbirth, early and late neonatal mortality were compared in mothers who were and were not referred. Factors associated with inter-institutional referral were analyzed using multivariable models with generalized estimating equations, adjusted for clustering at the level of the Primary Health Center.

**Results:**

Between June 2009 and June 2013, 3236 (9.4%) of 34,319 women had inter-institutional referral. Factors associated with referrals were maternal age (adjusted Relative Risk or aRR 1.1; 1.0–1.2); moderate or severe anemia (aRR 1.2; 1.2–1.4), gestational age <37 weeks (aRR 1.16; 1.05–1.27), multiple gestation (aRR 1.6; 1.2–2.1), absent fetal heart rate (aRR 1.7; 1.3–2.2), primigravida (aRR 1.4; 1.3, 1.6), primigravida with any pregnancy related maternal condition such as obstructed or prolonged labor; major antepartum or post-partum hemorrhage, hypertension or preeclampsia and breech, transverse or oblique lie (aRR 4.7; 3.8, 5.8), multigravida with any pregnancy related conditions (aRR 4.2; 3.4–5.2). Stillbirths, early neonatal,late neonatal and early infant deaths occurred in 7.3% referred mothers vs. 3.7% of not referred.

**Conclusions:**

Almost 10% of the women had an inter-institutional referral and still birth or neonatal deaths were doubled in referred women. Conditions associated with referral were often known before onset of labor and delivery. Improvements in maternal and neonatal outcomes will likely require pregnant women with conditions associated with referral to be directly admitted at facilities equipped to care for complicated pregnancies and at risk neonates, as well as prompt detection and transfer those who develop “at risk” conditions during labor and delivery.

**Trial registration:**

ClinicalTrials.gov NCT01073475.

## Background

The period around birth is critical for saving lives of mother and their infants. The large majority of maternal deaths (60%) and about half of stillbirths occurs during this intrapartum period. Roughly 45% of all under-five children’s deaths occur during the neonatal period. Of these 75% occur in the first week of life, half of which are on the first day of life due to intrapartum causes [1–3]. The majority of maternal deaths can be prevented by the presence of a skilled birth attendant who delivers high quality care and can access to an appropriate maternal referral system when needed [4]. In 2008, the Indian government introduced a conditional cash transfer (CCT) program, *Janani Suraksha Yojana* (JSY) that provides financial assistance for women delivering in institutions and to community health workers who bring the expectant mother to institutions for delivery. The JSY provides free treatment and delivery services to rural pregnant women. Financial incentives are provided to the community health worker (CHW) who accompanies and facilitates the delivery of the pregnant women at the health facility. This is a one time incentive to restrict home deliveries and encourage facility deliveries. It does not cover inter-institutional referrals. This scheme has substantially increased the rates of deliveries in facilities in India, [5] but to date, maternal mortality has not decreased, raising questions about the quality of care provided in facilities and access to a quality referral system when it is needed. Hallmarks of a quality referral system include: accurate screening, identification of women at risk, timely referral and transportation to a facility that can provide needed interventions and care, along with experienced and trained medical staff [6]. Such a referral system is so important that it has been called the keystone of safe motherhood [7, 8].

In India, the state government funded obstetric health system offers three levels of care in rural communities; primary, secondary, and tertiary. The tertiary level facilities provide specialized obstetric care along with allied medical specialty care. Secondary care is provided by district hospitals which have obstetric specialists available for caesarean sections. The primary health centers (PHC), situated in larger geographical rural settings, provide 24-h a day basic obstetric care including birthing facilities for vaginal deliveries and allied basic medical services. Sub-centers (SC) are birthing facilities with a trained birth attendant in the villages equipped only for vaginal deliveries. High risk pregnancies and those with intrapartum complications are eligible for referral from SCs, PHCs and secondary care to a suitable higher level of care. High quality care would ensure accurate and timely identification of at risk pregnancies and births and prompt referral of the woman and her baby to a higher level of care when at risk pregnancy or birth is detected.

As a first step to understanding where improvements need to be made to reduce maternal mortality and adverse pregnancy outcomes, we studied inter-institutional referrals of pregnant women admitted for labor and delivery in an ongoing Maternal and Newborn Health Registry in the rural area around Nagpur, Maharashtra. Our specific aims were to 1) determine the proportion of women who had an inter-institutional referral after admission for labor and delivery; 2) describe the pregnancy related maternal conditions requiring a higher level of obstetric care in women who had a referral versus those who were not referred; 3) report maternal, fetal and neonatal mortality in women who were referred versus not referred.

## Methods

### Study design, setting and participants

The Maternal Newborn Health Registry (MNHR) in Nagpur, India is part of a prospective multi-country cohort study of pregnant women and their babies. It is funded by the *Eunice Kennedy Shriver* National Institute of Child Health and Human Development (NICHD) Global Network for Women’s and Children’s Health Research. Pregnant women are recruited as early as possible during pregnancy and followed through day 42 post-partum to obtain details about the pregnancy, labor, delivery and the health of the mother and infant. At the Nagpur site, pregnant women are recruited from the rural communities that are in the catchment areas of 20 PHCs in 4 districts - Nagpur, Bhandara, Wardha and Chandrapur. The methods and initial data have been described in detail elsewhere [9].

**Fig. 1:**
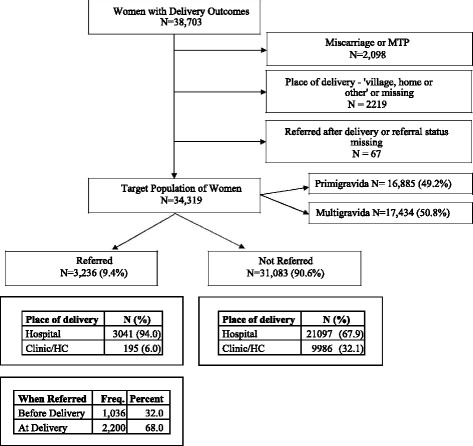
Flow diagram of pregnant women included in the maternal referrals study

For this study, we included pregnant women enrolled in the MNH Registry in Nagpur (Fig. 1). All pregnant women who are permanent residents of the catchment area were consented to enroll in the MNHR. The participation rate was 99%. We excluded women whose pregnancy ended in a miscarriage or a termination prior to labor and delivery, women who delivered at home, women who were referred to a second facility but information on the place of referral or date of hospitalization at the referral facility was missing. Since our goal was to study inter-institutional referral associated with labor and delivery, we excluded women who were referred after the day of delivery.

### Data source

Trained study staff obtained data by personal interviews of pregnant women and their hospital discharge cards, at 3 time points: (1) on enrolment, information is collected on the date of last menstrual period, estimated delivery date, age, education, and parity, (2) within 7 days of delivery, information is collected on complications occurring during pregnancy, referrals/ transfer to other institutes, details of labor and delivery, including place, mode of delivery, provider, actual birth weight obtained at the time of birth, status of the mother and newborn following delivery, and treatment provided to the mother and newborn at referral facilities; and (3) at 42 days postpartum, information on maternal and newborn health and status is assessed. *Inter-institutional referral* was defined as transfer of a woman from a first or second level facility to which she was admitted for labor and delivery to a facility providing higher level of care at any time after admission to the day of delivery. *Maternal Death* was defined as a death that occurred any time between admission for labor and delivery through day 42 post partum. A *Stillbirth* was defined as a baby having no signs of life at birth. *Early Neonatal Deaths* were those occurring during the first 7 days of life. *Late Neonatal/Early Infant Deaths* were those occurring between day 8 and 42 of life. We collected information on individual maternal characteristics at enrolment, and, maternal conditions that potentially required a higher level of obstetric care from hospital records within 7 days of delivery. The individual characteristics were: maternal age (<25 years, >25 years); parity (primigravida if the woman was pregnant for the first time with the fetus reaching beyond the age of viability (20 weeks) and multigravida if she was pregnant more than once with the fetus reaching beyond the age of viability); education (illiterate/primary, secondary or higher); maternal body mass index (BMI, <21, 21–30, >30); maternal anaemia (none > 11gm/dl, mild10 to 11 gm/dl, moderate/severe <10 gm/dl) [10]; multiple gestation (versus singleton pregnancy); and fetal heart rate detected before delivery. *Pregnancy related maternal conditions requiring a higher level of obstetric care* were presence of one or more of the following: obstructed/prolonged labor, major antepartum hemorrhage (APH), major post-partum hemorrhage (PPH), hypertension/pre-eclampsia, and breech, transverse or oblique lie. All data were entered centrally. Data audits, including inter- and intra-form consistency checks were performed at data entry, and additional audits were performed by the data centre (Research Triangle Institute, NC, USA).

### Statistical analyses

#### Descriptive statistics

Descriptive statistics were used to compare characteristics of mothers who were referred and those who were not referred. Maternal mortality was also calculated as a proportion of women admitted for labor and delivery. The stillbirth rate was calculated as proportion of all births and early neonatal mortality and late neonatal/early infant mortality were calculated as a proportion of all live births.

#### Univariate and multivariable analysis

We calculated the relative risk and 95% confidence interval for factors potentially associated with inter-institutional referral. Significant factors were included in a multivariable model with a Poisson distributional assumption and log link with maternal referrals as a response variable. Generalized Estimating Equation (GEE) extensions were used to account for correlation of outcomes within clusters (PHC). A multivariable model was used to obtain adjusted risk ratios with all risk factors included as covariates. An interaction term of any pregnancy related maternal conditions and parity was included in the multivariable analyses to generate estimates of each level of cross classification of these factors on maternal referral status. All analyses were performed in STATA 13.1 (Stata Corp. 2014. Stata Statistical Software: Release 13.1 MP4.StataCorp LP: College Station, TX, USA).

## Results

We studied 34,319 (89%) of the 38,703 women enrolled in the MNH Registry between June 2009 and June 2013. The reasons for excluding the remaining 11% are shown in Fig. 1. Forty-nine percent were primigravida women and 51% were multigravida women.

### Inter-institutional referral

Of the 34,319 women, 9.4% had an inter-institutional maternal referral, 68% of whom were referred to another facility on the day of delivery. Almost all mothers (99.7%) who were referred were transported to the referral facility within two hours of referral decision and 99.9% of them were seen within 2 h of reaching the hospital.

### Pregnancy related maternal conditions requiring a higher level of obstetric care

**Fig. 2:**
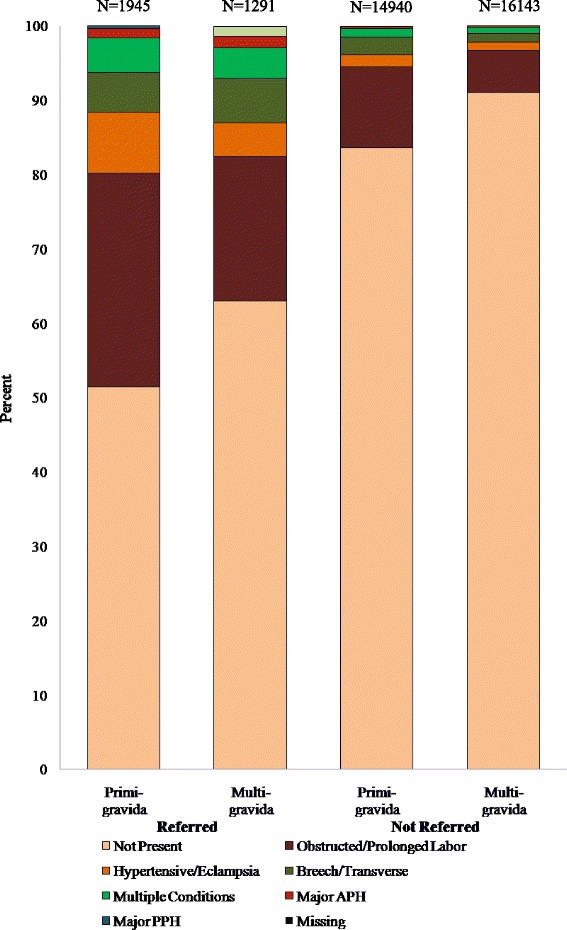
Maternal conditions in referred and non-referred women, by Parity

**Fig. 3:**
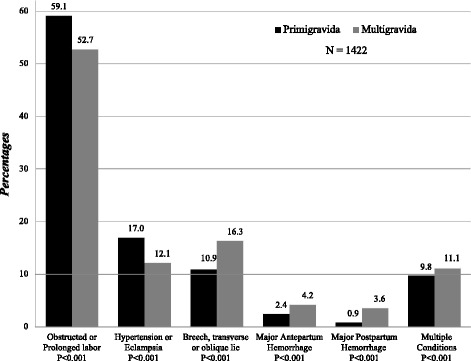
Proportions of referred women with reported pregnancy related maternal conditions, by Parity

Women who were referred were significantly more likely to have one or more pregnancy related maternal conditions versus those who were not referred (43.9% versus 12.4% respectively, *p* < 0.001), as shown in Table 1. Figure 2 shows a distribution of maternal conditions in women, by parity in women who were referred and those who were not referred. Of note, over half of the women who were referred did not have pregnancy related maternal conditions while 12% of the women who were not referred had pregnancy related maternal conditions. Figure 3 shows the paritywise distribution of the conditions in the women who were referred, with obstructed/prolonged labor being greater than three times more frequent than any another condition among women who were transferred. The rates of caesarian section in those who experienced inter-institutional transfers was 1127/3236 (35%) as compared to 5613/31083 (18%) in those who did not experience this transfer.

**Table 1:** Risk factors for maternal referrals, univariate and adjusted multivariable estimates

	Mother referred *N* = 34,319 (100)		Univariate	Multivariable
	Yes N (%)	No N (%)	RR (95%CI)	aRR (95%CI)^a^
	*n* = 3236 (9.4%)	*n* = 31,083 (90.6)		
Maternal Age (Years)				
< =25	2345 (72.5)	21,367 (68.7)	1.2*** (1.1–1.3)	1.1** (1.0–1.2)
25+	891 (27.5)	9716 (31.3)	Referent	Referent
Maternal Education				
Illiterate or Primary	592 (18.3)	6267 (20.2)	Referent	Referent
Secondary or Higher	2638 (81.5)	24,755 (79.6)	1.2*** (1.1–1.3)	1.1 (1.0–1.2)
Missing	6 (0.2)	61 (0.2)	–	–
Maternal BMI				
< 21	1966 (60.9)	19,461 (62.6)	0.9 (0.9–1.0)	1.0 (0.9–1.0)
21–30	1197 (37.0)	10,912 (35.1)	Referent	Referent
30+	73 (2.1)	710 (2.3)	0.9 (0.7–1.1)	0.9 (0.7–1.2)
Maternal Anemia				
None	300 (9.3)	2876 (9.3)	Referent	Referent
Mild	1359 (42.0)	13,074 (42.1)	0.9 (0.8–1.0)	1.1 (1.0–1.3)
Moderate or Severe	1525 (47.1)	14,617 (47.0)	1.0 (0.9–1.0)	1.2***(1.0–1.4)
Missing	52 (1.6)	516 (1.6)	–	–
Maternal Gestation				
(weeks)				
< 37 weeks	511 (15.8)	4193 (13.5)	1.2*** (1.1–1.3)	1.2***(1.1–1.3)
> = 37 weeks	2685 (83.0)	26,517 (85.3)	Referent	Referent
Missing	40 (1.2)	373 (1.2)	–	–
Multiple Gestations				
No	3181 (98.3)	30,874 (99.3)	Referent	Referent
Yes	55 (1.7)	209 (0.7)	2.1*** (1.6–2.8)	1.6***(1.2–2.1)
Fetal Heart Rate Present				
No	58 (1.8)	240 (0.8)	2.1*** (1.6–2.8)	1.7***(1.3–2.2)
Yes	3122 (96.5)	30,619 (98.5)	Referent	Referent
Missing	56 (1.7)	224 (0.7)	–	–
Interaction term				
Primigravida - No PRC^b^	1001 (30.9)	12,498 (40.2)	1.4***(1.3–1.6)	1.4***(1.3–1.6)
Primigravida - PRC	944 (29.2)	2437 (7.8)	5.0***(4.0–6.3)	4.7***(3.8–5.8)
Multigravida - No PRC	813 (25.1)	14,714 (47.4)	Referent	Referent
Multigravida - PRC	478 (14.8)	1428 (4.6)	4.4***(3.6–5.6)	4.2***(3.4–5.2)

**P < 0.05 ***P < 0.001

^a^Multivariable RR adjusted for 20 PHCs (clusters) using GEE Poisson regression

^b^One or more Pregnancy Related Conditions in mothers

Table 1 shows the univariate relative risk and multivariable adjusted relative risk for presence of any pregnancy related condition requiring a higher level of obstetric care and other potential factors associated with inter-institutional referral. In the multivariable model, in addition to presence of any pregnancy related maternal conditions, risk factors for upward referral included maternal age, moderate or severe anemia, gestational age <37 weeks, multiple gestation, absence of fetal heart rate before delivery, primigravida, primigravida with any pregnancy related maternal condition such as obstructed or prolonged labor; major antepartum or post-partum hemorrhage, hypertension or preeclampsia and breech, transverse or oblique lie, multigravida with any pregnancy related conditions Referrals were significantly more likely to occur in woman younger than 25 years.

### Maternal mortality, stillbirths, early neonatal deaths and late neonatal/early

#### Infant deaths

There were a total of 13 maternal deaths up to day 42 post-partum. One death occurred in a woman who was referred (1/3236; 0.03%) and was due to hemorrhage with cardio respiratory arrest. Twelve deaths occurred in women who were not referred (12/31,083; 0.04%). The causes of death in non-referred mothers were sepsis (2), pre-eclampsia/eclampsia (1), suicide (3), burns (2), accidental (1), brain hemorrhage (1), ischemic heart disease (1), and postnatal psychosis with cerebral malaria (1).

Stillbirth or neonatal death occurred in 216 (6.7%) of 3236 women referred and 715 (2.0%) of 31,083 women not referred (*p* < 0.001). Specifically, stillbirths occurred in 116 (3.6%) of women who were referred versus 548 (1.8%) of women those who were not (*p* < 0.001); early neonatal deaths occurred in 100 (3.1%) of those referred vs 466 (1.5%) of those not referred (*p* < 0.001); and late neonatal/early infant deaths occurred in 21 (0.6%) of the women who were referred versus 128 (0.4%) of those not referred (*p* < 0.001).

## Discussion

In the rural communities around Nagpur, Maharashtra, in Central India between 2009 and 2013, we found that almost 1 in 10 women admitted for labor and delivery were referred to another institution between admission and the day of delivery, mostly on the day of delivery. This result is not surprising since more than 50% of the women who were referred had obstructed or prolonged labor, and consequently higher rates of stillbirth, neonatal and maternal mortality, than those not referred. Our referral rates are similar to those previously reported by Chaturvedi et al., who also reported more adverse birth outcomes in women referred during labor and delivery [8].

Our findings raise several important concerns. It suggests that if 1 in 10 women need inter-institutional referral, then obstetric and neonatal care available at first level facilities (mostly birthing centers at PHCs or SCs equipped only to manage uncomplicated vaginal deliveries) is insufficient to meet obstetric needs of high risk women from rural communities. Outcomes for women referred are worse, likely because women who were referred were at higher risk of adverse outcomes due to known conditions prior to admission for labor and delivery or conditions that developed during labor and delivery. This suggests that women with known “at risk” conditions may benefit from direct admission to higher level facilities for labor and delivery, especially for primigravida women in whom the presence of pregnancy related conditions increased the risk of referral several folds. It is also likely that improvements in quality of care are needed in first level facilities to ensure timely recognition and prompt referral when “at risk” conditions develop in these facilities. Third, since the implementation of the financial incentive scheme for institutional deliveries in India in 2008, facility deliveries have increased from 47% in 2005–6 (National Family Health Survey-3) to more than 96% in Maharashtra in 2014 [11–13], but neither the maternal or neonatal mortality rates [14] have changed significantly. This raises questions about the quality of care in facilities in general and those women who are referred appear to be at high risk of adverse outcomes to themselves or their babies. There is an urgent need to evaluate quality of care across the spectrum of facilities where women deliver their babies and particularly timely recognition of need for maternal referral.

Our study may provide some guidance where improvements could be made. Those in premature labor or those with multiple gestations who need higher levels of care could be targeted for pre-labour admission to facilities that can provide experienced and trained medical staff who can provide needed interventions and care for small and premature babies. There is also a need for improvements in timely recognition of pregnancy related maternal conditions that develop during labor, such as obstructed/prolonged labor and hypertension in primigravida women and antepartum and postpartum hemorrhage in multigravida women. Earlier detection and referral of women with a fetus in breech, transverse or oblique lie may be beneficial to all women. There were 13 maternal deaths up to day 42 post-partum recorded from the MNHR during the study period. Out of these only 1 woman had experienced an inter-institutional referral. However her death occurred a month after delivery and the recorded cause of death was haemorrhage with cardio-respiratory arrest. The remaining 12 deaths occurred in woman who did not experience any upward inter-institutional referral and died many days after delivery.

A major strength of our study is that it is one of the first and largest prospective, population-based, descriptive studies of inter-institutional maternal referrals in Central India after the implementation of JSY that was specifically designed to increase institutional deliveries in India [5]. A second strength is the high quality data collection procedures that has detailed maternal and infant outcomes [9]. Limitations include the absence of some data about maternal referral and factors associated with referral, particularly delays in care-seeking behavior, or clinical decision making and measures of quality of care in facilities. We were also not able to assess the severity of the conditions present at birth or the level or quality of care at the referred facility. Our results would not be generalizable to other low and low/middle income countries that do not have schemes like JSY to encourage institutional deliveries.

## Conclusions

Rates of maternal referrals in the rural communities around Nagpur, Maharashtra, in Central India are high. Referral is associated with adverse outcomes for mothers and their babies. There is an urgent need for studies to assess and address quality of care at all levels of facilities that provide obstetric services. It would also be appropriate to prospectively investigate inter-institutional referral as a potentially new risk factor for adverse outcomes.

## Abbreviations

APH: Antepartum haemorrhage
aRR: Adjusted relative risk
CCT: Conditional cash transfer
CHW: Community health worker
GEE: Generalized estimating equations
IRB: Institutional Review Board
JSY: Jannani Suraksha Yojana
MNHR: Maternal and Newborn Health Registry
NICHD: *Eunice Kennedy Shriver* National Institute of Child Health and Human Development
PHC: Primary Health Center
PPH: Post partum haemorrhage
SC: Subcenter
